# Framework-mediated binding of foreign and self-glycans by IGHV4–34 antibodies

**DOI:** 10.3389/fimmu.2026.1767837

**Published:** 2026-02-18

**Authors:** David B. Langley, Christopher J. Jara, Jake Y. Henry, Joanne H. Reed, Christopher C. Goodnow, Daniel Christ

**Affiliations:** 1 Immune Biotherapies Program, Garvan Institute of Medical Research, Darlinghurst, NSW, Australia; 2 St. Vincent’s Clinical School, Faculty of Medicine, University of New South Wales Sydney, Sydney, NSW, Australia; 3 The Westmead Institute for Medical Research, The University of Sydney, Westmead, NSW, Australia; 4 Cellular Genomics Futures Institute, University of New South Wales Sydney, Sydney, NSW, Australia

**Keywords:** cold agglutinin autoantibodies, cold agglutinin disease, FR1 hydrophobic patch, glycan-antibody interface, IGHV4-34 antibodies

## Abstract

Cold agglutinin disease is an autoimmune condition characterised by expression of self-reactive antibodies to I/i carbohydrate blood group antigens (polymers of N-acetyllactosamine or ‘LacNAc’ units) resulting in anaemia through the agglutination and complement-mediated destruction of red blood cells. This antibody response is dominated by antibodies of the human IGHV4–34 germline, which display a unique hydrophobic patch formed by germline-encoded tryptophan and tyrosine residues at positions 7 and 25 within framework 1 (FR1). Although the requirement for conservation of this FR1 patch for binding to the I/i carbohydrate antigen has been well established, structural insights regarding the mechanistic role of the FR1 patch have remained elusive. Intriguingly, recent papers describing IGHV4–34 antibodies bound to the glycan-adorned surface of the HIV envelope glycoprotein shed light on the utility of this FR1 surface. Rather than indirectly shaping the conformation of the conventional antigen binding site as previously proposed, the structures reveal direct interfaces between the hydrophobic FR1 patch and high mannose glycans projected by HIV. Given the stereochemical similarities between these glycans and LacNAc, these structures suggest how I/i self-antigen glycans might be similarly engaged by IGHV4–34 antibodies in a framework-centric non-CDR mode and provide a rationale for the preservation of this otherwise self-reactive antibody germline.

## Introduction: CAD and CAS

Cold agglutinin disease (CAD) and cold agglutinin syndrome (CAS) are autoimmune conditions involving the dysregulated proliferation of monoclonal or polyclonal B cells, respectively, which produce antibodies of the IgM isotype directed against self I/i antigens on red blood cells (1–6). The affinity of cold agglutinins is generally low at core body temperature, but binding is enhanced in the peripheral circulation where the temperature drops below 32° C. When these IgM autoantibodies are present at sufficient concentration, the resulting agglutination of red blood cells obstructs small blood vessels while the binding of IgM fixes complement C1-C5 resulting in opsonisation, erythrocyte destruction and anaemia.

## Autoantigens I and i

**Figure 1:**
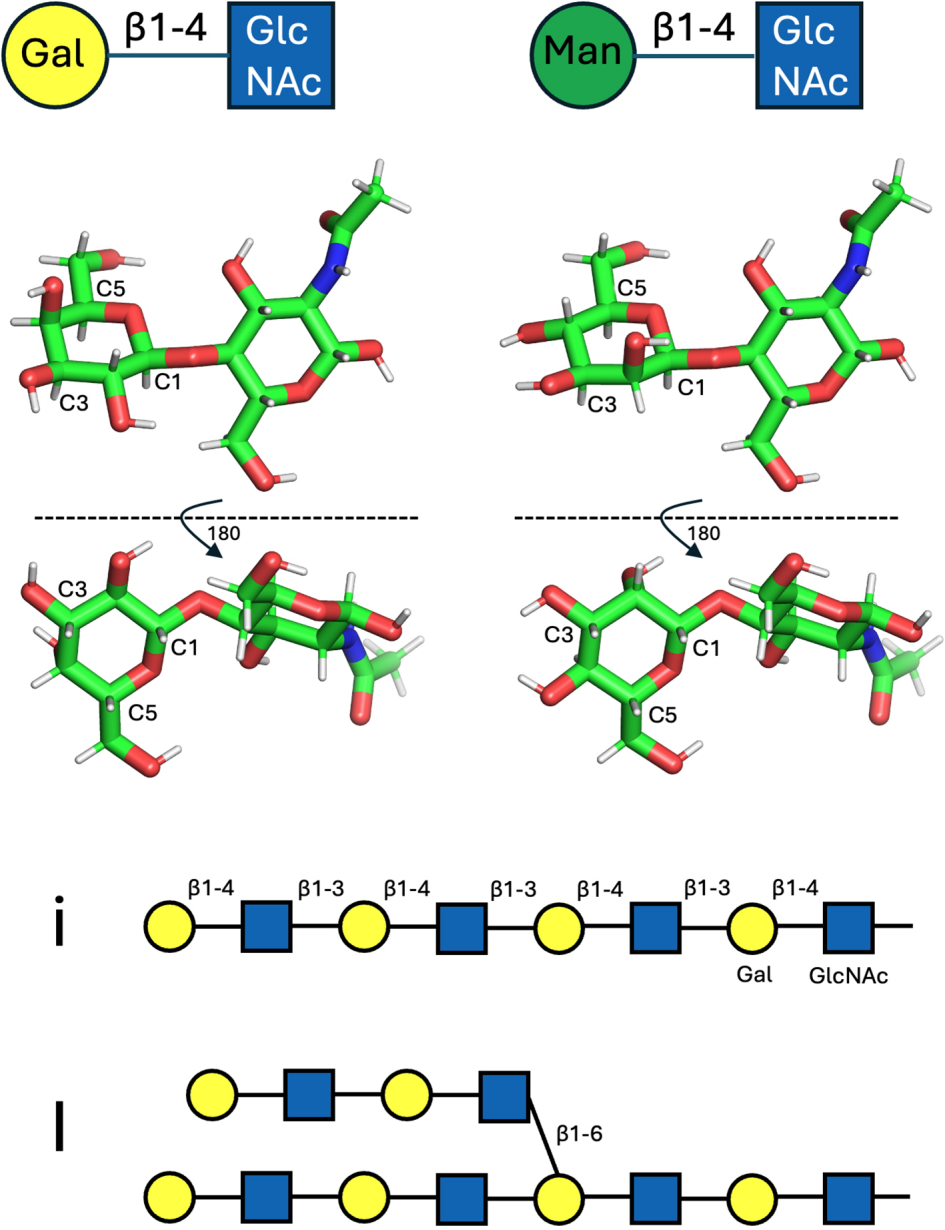
Glycan representations. Top: LacNAc (Galβ1-4GlcNAc) and Manβ1-4GlcNAc disaccharide units (LHS and RHS, respectively), as depicted by symbol nomenclature (top row) and as molecular sticks (middle, as viewed from two perspectives). The riding protons are shown as grey sticks. Both Gal and Man entities project a similar arrangement of riding protons off carbon positions C1, C3 and C5, which project from the same plane of the hexose rings. Bottom: symbol depiction of linear (i) and branched (I) forms of LacNAc polymers.

The existence of human blood group antigens was established as early as 1901 by Landsteiner (7), who also recognised that in some people ABO blood typing is complicated by the presence of low-titre serum cold agglutinins (8). However the molecular basis and carbohydrate (glycan) nature of the blood group antigens was only recognised in the mid-20^th^ century, with carbohydrates also being increasingly recognised as clinically important as onco-developmental markers and as underpinning many autoimmune conditions (9–11). In the mid-1950s Wiener et al., reported cold autoantibodies recognising a self-antigen termed ‘I’, for ‘individuality’ (3). In 1960 Marsh & Jenkins predicted that the alternate ‘i’ phenotype, dominant in cord blood, was not representative of an alternate allele of I, but a failure of epitope maturation to the adult (I) form (12, 13). Self-antigen entities i and I were subsequently revealed as linear (neonatal) and branched (adult) repeats of the disaccharide N-acetyllactosamine (LacNAc; Galβ1-4GlcNAc) (**Figure 1**, top left and middle left). The anti-i epitope comprises a linear repeat of LacNAc units connected in a head-to tail fashion via β1–3 linkages (14), while the I antigen refers to similar polymers containing branch points via β1–6 linkages (**Figure 1, bottom**) (15). Addition of the β1–6 linkages is attributed to the activity of I-branching β1-6-N-acetylglucosaminyl transferases (IGnTs), which become developmentally upregulated at around 9 months of age. Disruption of these IGnTs results in the ‘adult i’ phenotype (16–19). As well as being ubiquitously expressed on glycoproteins and glycolipids of many cell types, the LacNAc moiety is a building block of other carbohydrate entities and is thus frequently displayed cryptically in other contexts; for instance, in the ABO blood group system, α1–2 fucosylation of the Gal moiety of LacNAc yields the H backbone upon which the A- and B-antigens are formed (20). Although B cells presenting antibodies against self I/i epitopes persist in the immune repertoire of healthy individuals, expansion of these cells and secretion of pathogenic I/i autoantibodies can result in forms of cold autoimmune haemolytic anaemia; CAD due to monoclonal expansion of B cells acquiring somatic oncogenic mutations (6, 21), and CAS typically in concert with infection by bacteria such as Mycoplasma pneumoniae and viruses which adsorb and/or display self I/i carbohydrates (22).

## IGHV4–34 germline restriction

A common factor in CAD and CAS is that the proliferating B cells producing the autoantibodies are germline-restricted to the immunoglobulin heavy chain family IGHV4-34 (formerly VH4.21 or DP-63) (23–26), and further, that the framework 1 region (FR1) of this germline, as distinct from the classically important complementarity determining regions (CDRs), which form the canonical antigen binding site, might be key. An important tool allowing the identification of IGHV4–34 B cells and antibodies is the anti-idiotype (Id) rat antibody 9G4 (27), which specifically targets this FR1 region (28). Interestingly, 3-11% of naïve B cells in the follicles of lymph nodes and tonsils of healthy people were identified as 9G4+ (IGHV4-34), yet these cells account for only 0.2% of serum IgM (27, 29) and have low surface IgM, a classic metric indicating that they are functionally hyporesponsive or anergic (30). Recent analysis of IGHV4–34 knock-in mice confirm this conclusion (31).

Increased IGHV4-34-restricted (9G4+) B cells and secreted antibody are also associated with disease severity in systemic lupus erythematosus (SLE) (32–34), although with notable differences to CAD/CAS. Although SLE-related autoantibodies are often IGHV4–34 derived, the isotype is often switched from IgM to IgG (35). Additionally, the primary antigens are less-well defined and more varied, including nuclear constituents associated with apoptotic cells such as ssDNA and dsDNA (36, 37) with possible cross-talk and/or induction with lipid-A, a bi-phosphorylated β1-6-linked glucosamine disaccharide (with fatty acid tails), presented on the surface of infective bacteria (38). Furthermore, in SLE the CDRs of the canonical antigen binding sites are often expansive in both sequence and length and often include positively charged amino acids (36). Additionally, the distinctive FR1 regions have often been modified or can be mutated (hence 9G4-) without affecting binding to non-I/i autoantigens, demonstrating that in SLE, epitope binding is predominantly of a canonical CDR-mediated nature and independent of the 9G4+ FR1-centric idiotype (37).

## IGHV4–34 germline structure

**Figure 2:**
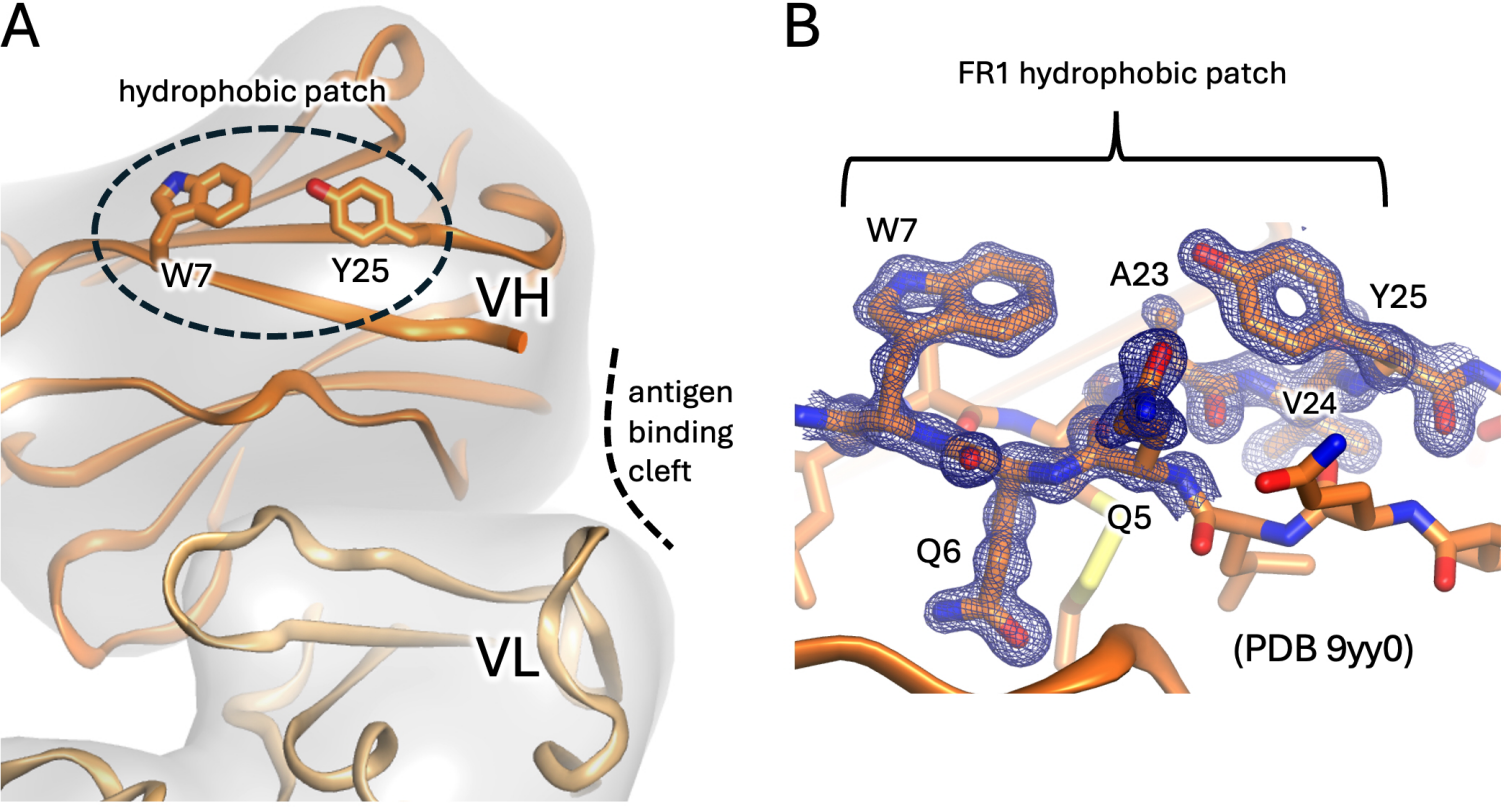
The FR1 hydrophobic patch features of IGHV4–34 antibodies. **(A)** Cartoon representation with heavy and light chains coloured dark and light orange, respectively. The framework 1 region (FR1) is indicated by the dashed sphere with residues W7 and Y25 shown as sticks and displayed on the surface. The hydrophobic patch is distinct from the canonical antibody antigen binding site (dashed curved line). **(B)** Close-up of hydrophobic patch from highest resolution IGHV4–34 antibody to date (antibody pre166, PDB entry 9yy0). The blue mesh depicts a composite omit map (2fobs-fcalc) of electron density at 1.8 sigma about the QQW and AVY sequence motifs.

The germline IGHV4–34 antibody is distinct from the other (approximately 70) human IGHV germlines by encoding a unique tryptophan (W) at position 7 (within the amino acid context QQW), as well as a tyrosine (Y) at position 25 (within the context AVY, implicated as being centric to 9G4 binding (28)). No other IGHV germlines encode bulky aromatic residues at these positions which, combined, form the unique FR1 region of IGHV4–34 antibodies. The structure of this FR1 region is exemplified by the structure of KAU - the first IGHV4–34 antibody to have its crystal structure solved (39) - in which the aromatic indole (W) and phenol (Y) sidechains of W7 and Y25 project into the bulk solvent, effectively adjacent and facing each other and forming a characteristic ‘hydrophobic patch’ on the surface (40) (**Figure 2**), distinct and physically separated from the CDRs and antigen-binding cleft. This FR1 hydrophobic patch of IGHV4–34 autoantibodies has been shown to be crucial for I/i binding, although CDR composition and, to a lesser extent, light chain selection also appear to play a role (4, 40–42). Despite strong evidence of the importance of the FR1 hydrophobic patch to the recognition of I/i targets by IGHV4–34 antibodies, no molecular mechanistic insights as to how or why have emerged thus far, apart from the early-proffered hypothesis that the FR1 hydrophobic patch might conformationally affect CDR conformations indirectly through medium-range allosteric interactions (41).

## Cataloguing IGHV4–34 structures

To investigate IGHV4-34-mediated I/i recognition, we examined all IGHV4–34 structures within the Protein Data Bank (PDB) (43). At the time of writing, the PDB contains some 248,000 experimentally determined structures, including several thousand antibody structures (44). We identified those entries containing IGHV4–34 lineage antibodies using a FR1 hydrophobic patch specific search string (“QQW(X)_15_AVY”), where ‘X’ denotes any amino acid, which identified 47 entries which reduced to 37 unique IGHV4–34 antibodies, starting chronologically with the aforementioned KAU entry in 2000 which comprised just the Fab arm with no antigen present (39). Two of the entries containing this sequence motif were not human but Rhesus macaque antibodies of the related IGHV4–73 germline lineage. An additional three IGHV4–34 germline antibodies were identified using search strings to accommodate mutations (at position 7 or 25) which diminish the hydrophobicity of FR1. These PDB entries contained mutations at position 25 (Y25N or Y25S) while retaining the unmutated W7. Of this IGHV4–34 ensemble (**Supplementary Table S1**) most have been deposited since 2020 and are centric to the fields of HIV or SARS-CoV-2 research, targeting the spike glycoproteins of the virions, and potentially containing new and untapped structural information. Hence, we analysed each structure in terms of whether the IGHV4–34 antibody was captured bound to its antigenic ligand, whether the ligand comprised peptide or carbohydrate, as well as the conformation of FR1 and residues W7 and Y25. Approximately half of the 40 unique antibodies have heavy chain CDR3 (HCDR3) lengths ≥ 20 amino acids (“long H3” in **Supplementary Table S1**), compared to the average of 14–15 reported for human HCDR3 sequences (45). Eleven of the antibodies were captured without ligands. Most of the structures comprise antibody Fab fragments bound to polypeptide targets in a canonical CDR-centric manner mediated through the antigen binding cleft, with little or no contribution from ancillary carbohydrates that sometimes project from a glycosylated target (standard interface, as indicated in **Supplementary Table S1**). Two of the structures, however, are strikingly different: both involve IGHV4–34 antibodies binding to HIV surface glycoprotein (Env or gp120/gp41) with both structures including significant carbohydrate contributions (46, 47). Prior to detailed discussion of these structures, overviews on the peculiarities of protein-carbohydrate interactions (including CH-pi interfaces and the importance of tryptophan) and the relevance of affinity and avidity to glycan binding, are presented in **Box 1** and **Box 2**.

Box 1 **Protein-carbohydrate interfaces, carbon-pi bonds, and tryptophan.**

**Figure 3:**
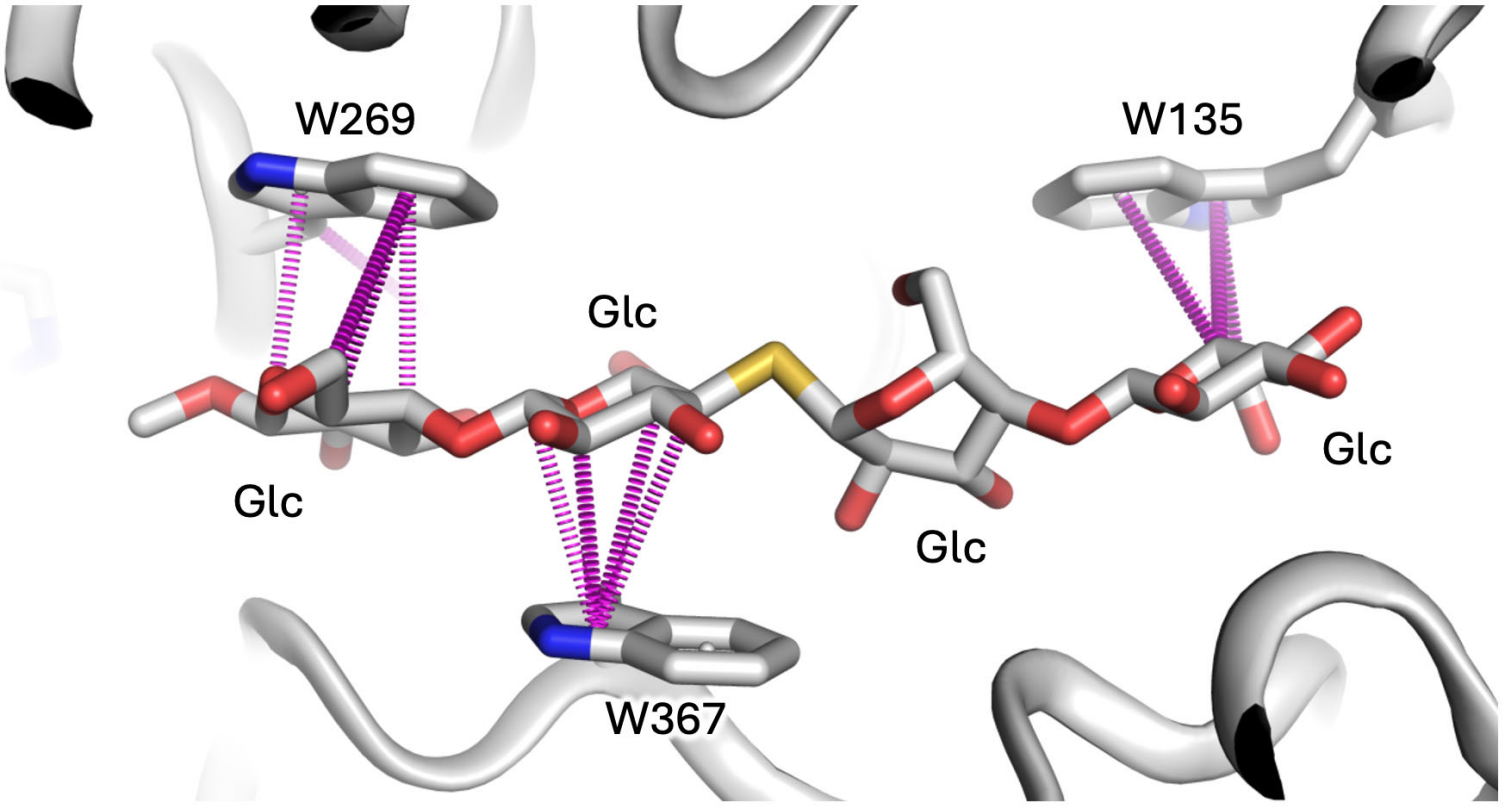
The importance of tryptophan to glycan interfaces is exemplified by the glycan-binding tunnel of cellulase Ce6A from Trichoderma reesei (PDB entry 1qk2) which contains three tryptophan residues that help position substrate (in this case a nonhydrolyzable sulphur-containing analog) by employing a network of CH-pi bonds (purple dashes) between the carbons of glucose moieties (projecting slight positive charge) and the centroids of the tryptophan rings (projecting slight negative charge). Image prepared using Arpeggio software.

Proteins typically bind other proteins by employing a mixture of hydrophobic, hydrophilic and charge complementarity: squeezing out solvent to allow hydrophobic surfaces to coalesce, connecting hydrophilic surfaces via hydrogen bonds, or juxtaposing positive and negative charges. Carbohydrates, however, being heavily hydroxylated compounds, lack substantial hydrophobic surfaces and as such don’t employ large-scale use of classical hydrophobic shape complementarity. None-the-less they still engage heavily with the aromatic subset of hydrophobic amino acid residues by way of carbon-pi (CH-pi or CH-π) interactions, as reviewed by Kiessling and Diehl (48). In short, due to the electronegative nature of oxygen atoms within the hydroxyl groups of sugars, the carbon atoms from which they project can be viewed as slightly electron-deficient and carry fractional positive charge. Further, every carbon within the ring of a furanose or pyranose heterocycle sugar unit caries a riding proton (by association also fractionally positive in charge) which is directed above or below the plane of the ring, depending on the stereochemistry and anomeric form of the sugar (as shown in **Figure 1, middle**). In some sugars (such as α-mannose or α-glucose) the distribution of hydroxyl groups, and thus CH-riding protons, above and below the plane of the sugar ring is mixed. In others, such as β-galactose or β-mannose (**Figure 1, middle**, LHS and RHS, respectively), one side of the ring projects an unencumbered cluster of three (or more) similarly projected CH groups (essentially a cluster of slight positive charge). Consequently, the CH-dense face of such sugar moieties is routinely found in protein structures stacked against the planar face of the side chains of histidine, tyrosine and tryptophan residues (see **Figure 3** for examples involving tryptophan). The heterocyclic side chains of these amino acids carry a delocalised electronic pi (π) system, with a slight negative charge above (and below) the plane of the ring. Hence, these aromatic rings form interactions with the positive CH clusters projected from carbohydrate moieties towards them, thus defining the CH-pi bond, which employ a mixture of forces both dispersive (London) and electrostatic in nature (49). Such CH-pi interfaces are frequently employed by carbohydrate-binding proteins to the extent that, statistically, the distribution of amino acids histidine, tyrosine and tryptophan are overrepresented in carbohydrate-binding pockets to the tune of approximately 2-, 3- and 9-fold, respectively, although phenylalanine bucks this trend and is not overrepresented (50). Tryptophan appears especially important, and in glycoside hydrolases, for instance, active site binding pockets frequently contain multiple tryptophan residues. For example, in the cellulase Cel6A from Trichoderma reesei, the binding tunnel is sequentially lined by 3 tryptophan residues to help position extended linear carbohydrate substrate [**Figure 3** (51)], while in the N-acetylglucosaminidase GcnA from Streptococcus gordonii, the active site pocket resembles a molecular cage lined on four of five sides by tryptophan side chains [PDB entry 2epn (52)].

Box 2 **Affinity and avidity.**

Estimates of individual hydrogen bond contributions to protein stability vary wildly depending on context, but on average they are believed to be in the order of 1 kcal/mol (53). Recent analysis of β-galactose containing PDB entries finds that each CH-pi interface contributes on average ~6 kcal/mol, while hydrogen bonds within this same dataset contribute ~4 kcal/mol, emphasising that individual CH-pi interfaces are at least, if not more, energetically important than individual hydrogen bonds to glycan-binding interfaces (49). As impressive as these metrics might seem, CH-pi interfaces are sterically larger than a simple hydrogen bond and are thus fewer; typically, one aromatic side chain per sugar unit. As well as lacking the hydrophobic surface complementarity common to protein-protein interfaces, interfaces involving carbohydrates also incur entropic penalties in the stripping and rearrangement of solvent molecules from the many hydroxyl groups projecting from sugars. Combined, the specificity and affinity between proteins and carbohydrates is relatively weak compared to protein-protein interfaces, with equilibrium binding constants typically in the order of only 1 μM, or worse, and orders of magnitude weaker than those of some protein-protein interfaces. To counter this, dedicated carbohydrate binders such as lectins often resort to avidity and employ multiple sugar-binding domains, sequentially arranged with each binding a carbohydrate target. Carbohydrates typically also oblige, with epitopes, such as I/i, presented as repeating units extending from the surfaces of proteins or cells (**Figure 1, bottom**).

## IGHV4–34 antibody ACS114

The first structure of interest was recently reported by Jelle van Schooten et al. (47), who isolated broadly neutralising antibodies (bnAbs) from memory B cells at different timepoints during HIV infection from a patient classified as an ‘elite virus neutraliser’. The bnAbs were categorised based on their germline lineages and the HIV surface glycoprotein (gp120/gp41) epitopes they targeted. One cluster comprised 7 clonally related antibodies derived from IGHV4-34, joining another distinct clone also derived from IGHV4-34. Intriguingly, single particle negative-stain electron microscopy (EM) confirmed that both of these IGHV4–34 antibody lineages targeted the same carbohydrate-heavy ‘silent face’ of gp120, an epitope heavily obscured by carbohydrate and so named to indicate being less accessible to antibody binding (54–56). One of the antibodies (ACS114; one of the 7 clonally related antibodies) subsequently had its structure solved by cryo-EM bound to glycosylated gp120/gp41 spike trimer (PDB entry 7zlk) (47).

**Figure 4:**
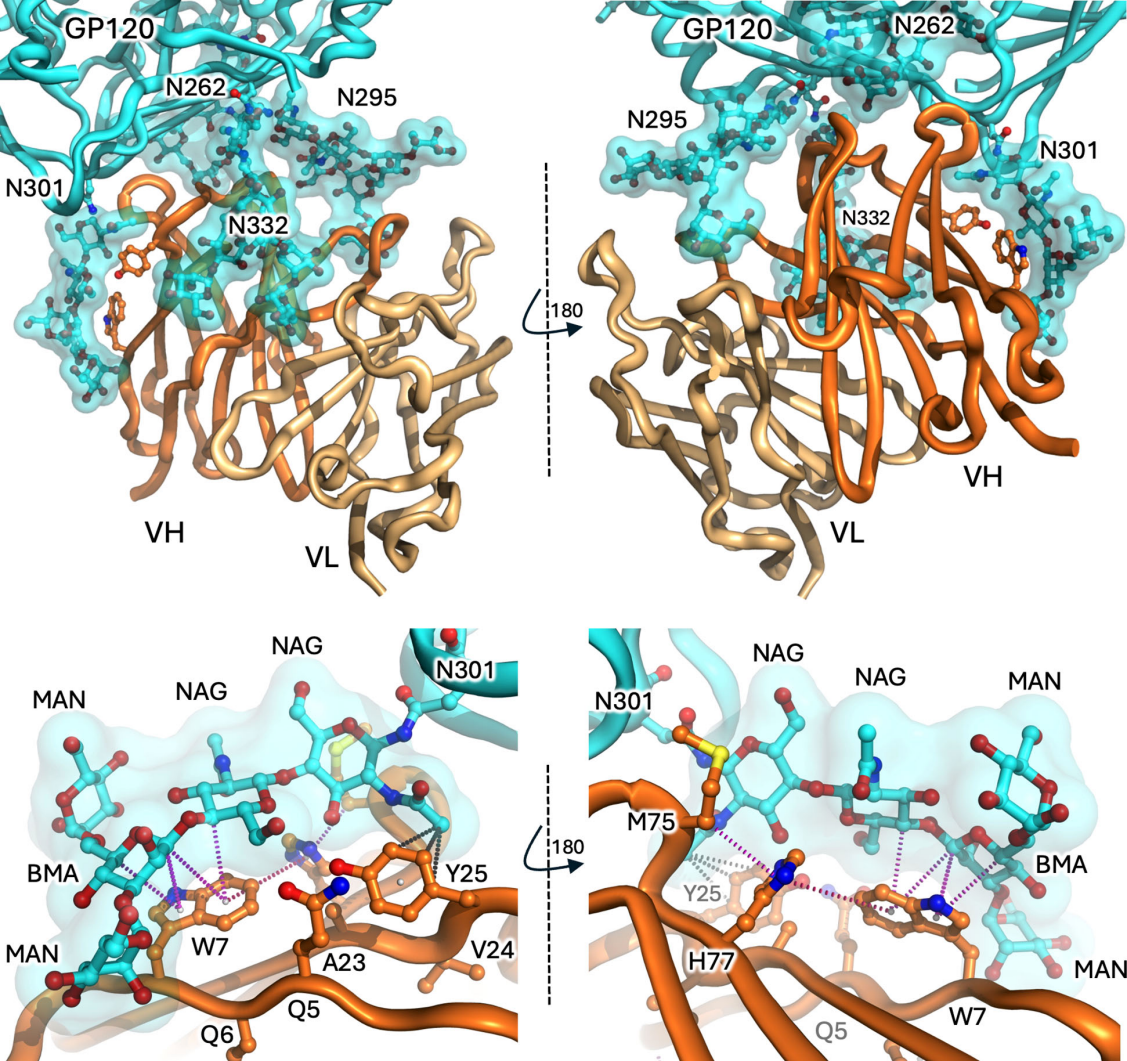
The IGHV4–34 antibody ACS114-GP120 interface. Top panels. The heavy and light chains of ACS114 (dark and light orange, respectively) interact with GP120 (cyan features) predominantly via four N-linked glycans (cyan surface and sticks). IGHV4–34 positions W7 and Y25 are shown as sticks. Two perspectives are shown. Bottom panels. Close-up perspectives of the interface between the hydrophobic surface FR1 region of the ACS114 heavy chain (particularly W7 and Y25) and the GP120 glycan projecting from N301. Potential CH-pi interfaces between CH atoms within the carbohydrate rings of the NAG and BMA entities and the ring centres of the tryptophan indole ring are shown as magenta dots. A ring-face interaction between the indole of W7 and imidazole moiety of H77 is shown as pink dots (likely stabilising the W7 rotamer) and possible hydrophobic interfaces involving Y25 are shown as grey dots. Interfaces predicted using Arpeggio analysis suite.

The structure reveals that approximately 90% of the antibody-gp120 interface is via the heavy chain of ACS114, with around 85% of the total interface formed by interactions with glycans (rather than protein) projected from four different asparagine (Asn; N)-linked positions on gp120 (glycans N262, N295, N301, N332) (**Figure 4**, top panels). The light chain of ACS114 makes no contact at all with the gp120 polypeptide, making only minor contact with the tip of glycans extending from N295 and N332. One of the glycans, projecting from N301, is draped over the IGHV4–34 hydrophobic patch, particularly over the side chain of W7. Contact analysis with Arpeggio software (57) reveals a network of CH-pi interfaces between the glycan and the W7 sidechain (**Figure 4** bottom panels, **Box 1**). The glycan projected from N301 is not poly-LacNAc, but a biantennary mannose (Man) core structure, as are those projected from N262, N295, and N332, although in the case of the N295 and N332-linked glycans, the core is extended with additional mannose sugars (unprocessed oligomannose subtype, typical for the Env protein (58)) which contribute to additional heavy chain, and minor light chain surface coverage. Despite the mannose-core:LacNAc difference, the CH-rich faces of β-mannose and β-galactose are chemically closely related (**Figure 1**, top, middle). In the ACS114 structure the branched mannose (BMA) is positioned over the tryptophan sidechain and is also coupled via a β1–4 linkage to GlcNAc, analogous to the coupling of β-galactose to GlcNac in the LacNAc repeats of I/i antigen (each LacNAc unit comprising Galβ1-4GlcNAc). Given chemical similarities, the BMAβ1-4GlcNAc contacting W7 of ACS114 might be reasonably viewed as a surrogate for how a LacNAc moiety within an I/i poly-LacNAc chain might interact with a IGHV4–34 hydrophobic patch (**Figure 4**, bottom panels).

This spike-antibody structure is somewhat complex – in addition to the spike trimer projecting three ACS114 Fabs (only Fv portions are modelled), two additional non-IGHV4–34 antibody Fabs (ACS122) project from another surface and break the pseudo-3-fold symmetry of the complex. In any case, the trimeric nature of the spike facilitates three views of the gp120-ACS114 interface for examination. Superposition about the gp120/gp41 components reveals only minor variations in glycan positions (mostly with terminal mannose residues projecting from N295 and N332), but the overall disposition of glycans with respect to the ACS114 antibody components are remarkably similar (**Supplementary Figure S1**). That being the case, we point out that the interface described is from a low resolution (4 Å) cryo-EM model, with the possibility that the hydrophobic patch rotamers have been modelled imprecisely or incorrectly, impacting the exact CH-pi features of the interface, and that one should be mindful of overinterpretation. That said, there is no question that the N301 glycan interacts in some manner with the FR1 hydrophobic patch (particularly W7) of the IGHV4–34 antibody.

Aside from the glycan-FR1 hydrophobic patch interaction detailed above, the other striking feature of the structure is the extent to which the antibody is contacted by multiple glycan ‘fingers’ projecting from gp120 (**Figure 4**, top panels). In conventional antibody-glycan interfaces the antigen-binding CDRs form some manner of cleft and envelope the carbohydrate target (59, 60). In this structure, however, the roles of antigen and antibody are somewhat reversed with the antibody heavy chain itself partially engulfed by glycan fingers projected from gp120 (akin to the webbing of a baseball glove holding a ball). The four carbohydrate fingers on their own each bury modest heavy chain surface area (on average N262, ~165 Å^2^; N295, ~335 Å^2^; N301, ~385 Å^2^; and N332, ~305 Å^2^) as well as limited light chain surface area (N295, ~100 Å^2^ and N332, ~60 Å^2^), as calculated using PDBePISA (61). A contact analysis of these other fingers reveals limited categorised interfaces (no other notable CH-pi interfaces and only a handful of possible hydrogen bonds), suggesting that the energetic contributions of each glycan finger towards binding affinity is likely to be limited. Strikingly, when one of these fingers was removed via an alanine substitution (N295A), neutralisation efficiency mediated by ACS114 was depleted by around three orders of magnitude (47). Even though the N295 finger is likely the most important – contacting both heavy and light chain CDRs – this finding suggests that avidity by way of contact with multiple glycan fingers might be crucial to this glycan-dominant antibody-antigen interface (see **Box 2**).

## IGHV4–34 antibody F6

**Figure 5:**
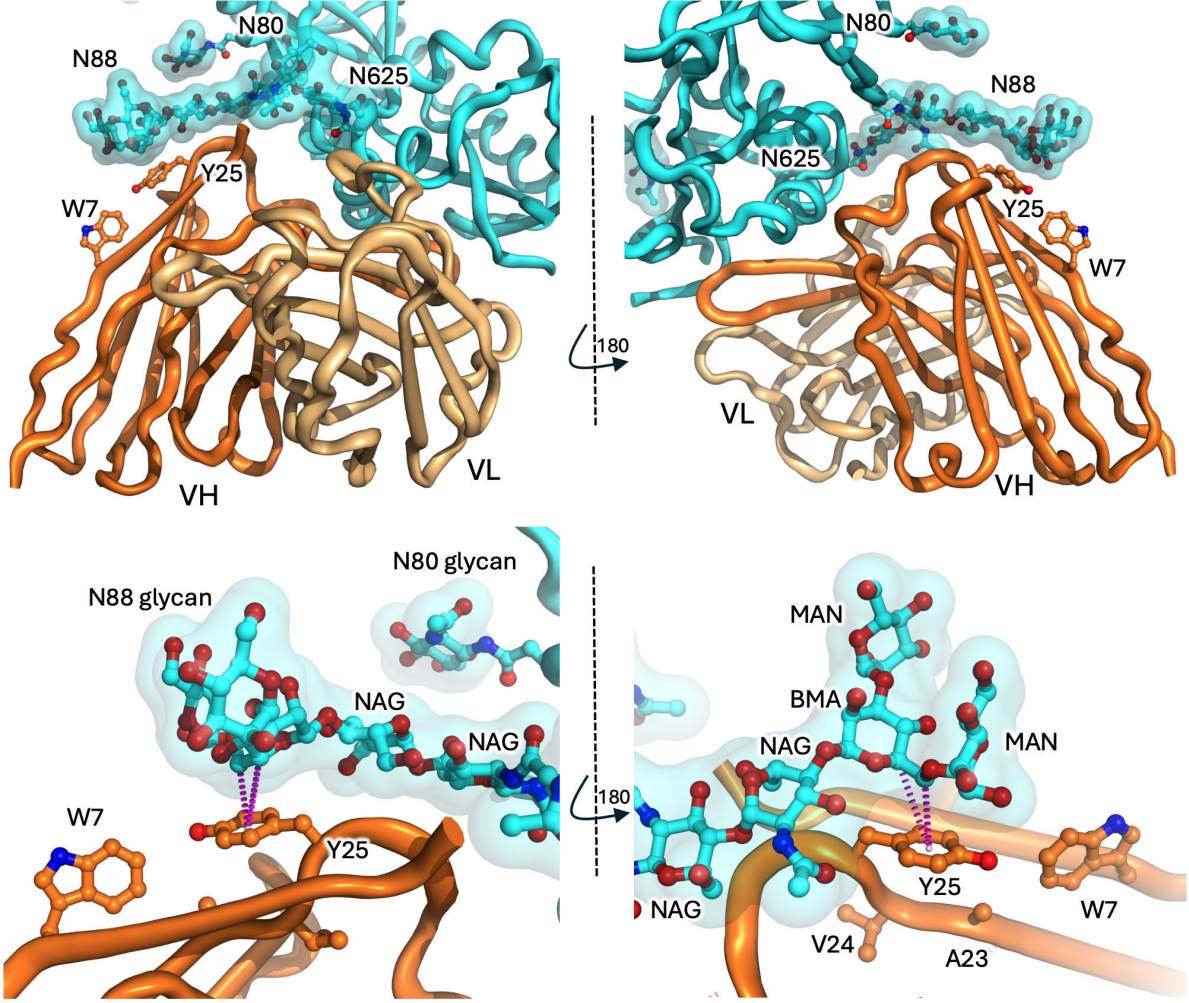
The IGHV4–34 antibody F6-GP120 interface. Top panels. The heavy and light chains of F6 (dark and light orange cartoons, respectively) interact with GP120 (cyan features) including via N-linked glycans (cyan surface and sticks). IGHV4–34 positions W7 and Y25 are shown as sticks. Two perspectives are shown. Bottom panels. The glycan projecting from N88 covers the Y25 position with the IGHV domain of F6. Potential CH-pi interface between CH atoms within the carbohydrate ring of the BMA and the ring centre of the tyrosine ring is shown as magenta dots (predicted using Arpeggio analysis suite).

A second IGHV4–34 antibody structure of significance due to an interesting carbohydrate interface was recently reported by Niu et al., who present the cryo-EM structure of antibody F6 complexing the HIV gp120/gp41 spike trimer (46) (PDB entries 8gpg at 4.10 Å (spike trimer) and 8gp5 at 4.05 Å (protomer local refinement)). The epitope targeted by F6 is not the silent face but a surface spanning the gp120/gp41 interface which is relatively unprotected by glycans. F6 binds in a more conventional CDR-centric manner, dominated by a prominent expanded HCDR3 loop (18 amino acids in length) that complements a surface cleft at the interface of gp120 and gp41 (**Figure 5**, top panels). The antibody heavy and light chains bury approximately 700 Å^2^ and 325 Å^2^ of target surface, respectively. However, the binding epitope is adjacent a mannose core glycan projecting from N88 of gp120, which contacts the AVY motif of FR1 (centred on tyrosine 25; Y25), burying an additional ~325 Å^2^ of heavy chain, although not extending to W7. Contact analysis (using Arpeggio (57)) suggests a CH-pi interface involving the BMA carbohydrate and sidechain of Y25 (**Figure 5**, bottom panels), although the same caveats with regards interpretation relating to structure resolution and the correct modelling of side chain rotamers apply (as for the ACS114 structure). Interestingly, while amino acid substitutions targeted to disrupt the antibody-polypeptide:gp120-polypeptide interface dramatically impacted equilibrium binding affinity, removal of the glycan projected from N88 glycan (N88A substitution) had little overall effect (simultaneously enhancing both the on- and off- rates ~2-fold, effectively cancelling each other out with regards overall affinity), with the authors suggesting that the N88 glycan likely acts as a ‘clasp’ to help preserve binding once the antibody is in place (46). However, the consequence of gp120 N88A mutation was not evaluated with regards F6 neutralisation breadth, where the additional glycan docking site on the hydrophobic patch may be pivotal for the capacity of the F6 antibody to neutralise HIV strains accumulating diverse mutations in the gp120-gp41 epitope. In any case, this is the only glycan interface we are aware of involving the AVY component of FR1 in an IGHV4–34 antibody and, combined, the structures involving antibodies ACS114 and F6 provide explicit structural evidence for the capacity of both W7 and Y25 residues within the FR1 hydrophobic patch to engage with glycans in an alternate framework-mediated manner that is both distinct from and distant from the canonical antibody-antigen binding site.

## IGHV4–34 germline response to infection

While CAD is predominantly a monoclonal lymphoproliferative autoimmune disorder, CAS is generally associated with heterogenous expansions and agglutinin production in response to either infection or the emergence of other malignancies (6, 62). IGHV4–34 responses to infection have been documented involving Mycoplasmas pneumoniae (63), Epstein Barr virus (EBV) (64), vaccinia virus (65, 66), HIV (67, 68) and more recently COVID19 where an assortment of case studies have linked SARS-CoV-2 infection to CAS (69–72).

In the case of vaccinia virus, our previous analysis of IGHV4–34 antibodies 166 and 589 revealed that their unmutated ancestors (pre166 and pre589) maintained bispecificity against the virus and I/i antigen presented on human lymphocytes (65). As a product of vaccination with smallpox and boosting with vaccinia virus three decades later, these antibodies acquired numerous V-region mutations, including some in the IGHV4–34 hydrophobic patch that eliminated binding to self I/i-antigen without compromising binding to the virus. In the case of 166, the antibody accrued the Y25S mutation (a key residue of the FR1 hydrophobic patch), while the 589 antibody accrued the Q6E mutation (directly adjacent W7), which has been shown to alter and destabilize the FR1region (73–75). As the matured 166 and 589 antibodies recognise non-glycosylated virus proteins there appears to be no selective advantage in maintenance of the self-glycan binding patch in FR1, and the loss of self-reactivity through hypermutation during infection is consistent with the clonal redemption mechanism of self-tolerance (65, 76–78).

Immunological responses to HIV and SARS-CoV-2 have been particularly intensely investigated, both of which are known to employ heavy glycan shielding, ostensibly in efforts to evade immunity-related attention from the host (79, 80). The HIV env trimer is particularly heavily camouflaged by glycans, which represent ~50% of the mass of the complex (81). Some surfaces, such as the silent face are especially cloaked in glycans and antibodies targeting this surface under-represented. In the case of the SARS-CoV-2 spike trimer, relatively fewer glycosylation sites exist although most appear to carry glycans (82), which proportionately constitute ~20% of the spike trimer mass (80, 83), sufficient to blanket a large proportion of the surface. Prior to the COVID19 pandemic it was reported that the IGHV4–34 response was dominant in the related MERS (84). Similar observations have been made for SARS-CoV-2, where the IGHV4–34 response was found to be overrepresented both in patients (85) and in response to immunisation with inactivated SARS-CoV-2 (86). Further, this immunisation study showed that progressive booster vaccinations correlated with an increase in HCDR3 length, implying that after initial interactions, possibly involving low-affinity glycan interactions, germinal centre maturation via somatic hypermutation accompanies a higher affinity response with an emphasis on CDR length to facilitate glycan-shield penetration.

## Parallels between IGHV4–34 and IGHV1–69 restriction

Just as a IGHV4–34 germline restriction has been noted in response to certain pathogens, a strong bias for B cells of the IGHV1–69 germline has been noted for viral insults including influenza (87) (targeting the hemagglutinin stalk), HIV (88, 89) (targeting gp41) and hepatitis C (88–90) (targeting the E2 envelope protein). IGHV1–69 antibodies are particularly noted for targeting epitopes with hydrophobic crevices, complemented by their projection of consecutive hydrophobic residues isoleucine (I) and phenylalanine (F), the ‘IF motif’, from the tip of HCDR2 (89). Although mature IGHV1-69-derived antibodies are often characterised by largely expanded HCDR3 loops (in the case of HIV to help breach glycan shields), initial attraction of the antibody class to specific targets is mediated by attraction between the IF motif and a hydrophobic pocket. Interestingly, this motif has also been demonstrated to be polyreactive and autoreactive against a spectrum of self-antigens, from dsDNA to phospholipids and cardiolipin (91), and even against the Fc domains of IgG antibodies, where the germline is coincidentally centric to an another autoimmune temperature-sensitive phenomenon distinct from CAD/CAS. In cryoglobulinemia, IGHV1–69 antibodies of mostly IgM isotype target the Fc regions of IgG antibodies resulting in the precipitation of immune complexes, particularly in acral appendages, resulting in the vasculitis associated with rheumatoid arthritis (92, 93), hence these antibodies being described as rheumatoid factors (RF). The IF motif at the apex of HCDR2 has proved central to this phenomenon, binding a conserved hydrophobic pocket on the surface of the Fc domain of IgG molecules (94).

## The IGHV4–34 self-reactive conundrum

IGHV4–34 germline antibodies have long been known to be self-reactive and centric to an assortment of autoimmune conditions, raising the evolutionary question of why the germline has been maintained. As evidenced by the structures described here, their capacity to grapple with glycosylated invaders is likely justification for their ongoing maintenance, despite the collateral potential for self-harm. That the germline is generally over-represented within circulating B cells speaks not only to their role in defence against glycosylated pathogens but also highlights the importance of the sophisticated tolerance checkpoint mechanisms that maintain IGHV4–34 B cells in an anergic state. Once sufficiently challenged to be coaxed out of anergy and progressed through somatic hypermutation and selection, the IGHV4–34 FR1 region central to autoimmune pathologies can either be maintained or mutated such that it is no longer I/i-autoreactive, as is the case with the mature 166 and 589 antibodies targeting vaccinia virus and is often the case with IGHV4-34-derived B cells noted in SLE. Among the IGHV4–34 antibodies structurally characterised, three of 40 entries (~ 8%) also have one of the two critical hydrophobic patch FR1 residues mutated (Y25N or Y25S, PDB entries shaded grey in **Supplementary Table S1**), presumably, at least to some extent, ‘redeeming’ such clones from potential hydrophobic patch-related autoreactivity. Unlike such redeemed antibodies, the HIV antibodies ACS114 and F6 retain an unmutated FR1 hydrophobic patch despite having acquired extraordinary numbers of V-region mutations during chronic HIV infection over many years. The structures of these antibody-HIV complexes uniquely reveal glycans as being intimately involved in contacting the FR1 hydrophobic patch. In the case of F6 the bulk of contact with HIV is via a conventional CDR-driven protein-protein interface, with the glycan-FR1 interface (involving Y25) being a minor contact (46). In contrast, the ACS114-gp120/gp41 structure is especially remarkable with the tip of the antibody arm, particularly the heavy chain component, being engulfed by an ensemble of glycans projecting off gp120 from four different N-linked positions, where one of these makes intimate contact with W7 of the FR1 hydrophobic patch (47). With little direct protein contact from the heavy chain domain (and none from the light chain domain), the glycan-dominated interface likely depends upon the avidity provided by contact with 4 non-overlapping glycan fingers, with removal of just one of these shown to be severely damaging to the neutralisation potential of the antibody. Clearly in these cases the disadvantage of self-reactivity conferred by the hydrophobic patch characteristic of the IGHV4–34 germline appears offset by providing an additional binding site, mediated by the same hydrophobic patch, for N-linked glycans on the HIV envelope glycoprotein.

The phenomenon of a matured antibody maintaining or developing inadvertent autoreactive qualities has been reported not just for the IGHV4–34 and IGHV1–69 germlines, but also for other IGHV elements. For instance, in the well documented maturation of the anti-HIV bnAb CH103, which targets the CD4-binding surface of gp120, the unmutated ancestor (IGHV4-59) started with polyreactivity against self-antigens that triggered B cell anergy, and during ongoing chronic infection antibody hypermutation increased this self-polyreactivity in concert with increasing virus neutralisation breadth (95, 96), thus highlighting the anergy-associated challenges associated with vaccine design and responses to targets such as HIV. Incidentally, antibody CH103 is additionally of interest as it also acquired framework 3 (FR3) mutations that enhance binding to the HIV trimer by facilitating intimate contact not only with one gp120 protomer epitope via canonical CDR-centric contacts, but also makes ancillary contact with an adjacent gp120 protomer via these FR3 substitutions (enhancing neutralisation by contacting two of the three gp120 protomers within the env trimer) (97), thus echoing the utilisation of framework surfaces to enhance binding to targets noted in IGHV4–34 antibodies.

## Summary

B cells of the IGHV4–34 lineage have long been known to be proportionately well represented relative to other germlines but are also known to be self-reactive and central to temperature sensitive autoreactive phenomena such as CAD and CAS, both believed to involve an IgM clumping of I/i antigens on red blood cells, leading to cell lysis and anaemia. Perhaps for this reason, IGHV4–34 cells show the markers of being maintained in a state of suppression or anergy (27, 29, 30). Collectively the structures of IGHV4–34 antibodies ACS114 and F6 (46, 47) give a tantalising glimpse of how the enigmatic FR1 hydrophobic patch involving W7 and Y25 might interface with poly-LacNAc chains (I/i antigen) on the surface of erythrocytes; the FR1 hydrophobic patch likely helps foster the tangling of IGHV4–34 antibodies with projections of carbohydrate, prompting low-affinity high-avidity engagement. Subsequent fine-tuning of specificity and affinity might be mediated by conventional HCDR modification and light chain selection. This basic thesis of IGHV4–34 engagement with I/i complements conjecture from 25–30 years ago (39, 41). It was supposed that FR1 hydrophobic properties were key, reflecting observations that the great variability in HCDR3 sequences effectively ruled out primacy of a conventional CDR-centric interface, with CDR and light chain contributions acting more to fine tune the dominant FR1 hydrophobic patch contribution. Although mechanistically it was suggested that the hydrophobic patch might in some way distort the conformation of the nearby conventional CDR-centric antigen binding site (39), with the structures currently available we see no evidence to support intramolecular distortion of the canonical CDRs and view the hydrophobic patch as an interface for low affinity glycan association, the capacity for which is inferred from the viral complexes herein described. We imagine that this propensity for low affinity glycan association might be how many pathogens are initially engaged by IGHV4–34 B cells. Early in an IGHV4–34 response the hydrophobic patch might help establish a foothold on heavily glycosylated targets, correlating mechanistically with how IGHV4–34 antibodies might naturally associate with self poly-LacNAc, followed by somatic hypermutation of HCDR3 and light chain selection to better penetrate the glycan shields of a pathogenic target. Interestingly, both ACS114 and F6 target the HIV env protein, renowned for employing heavy glycosylation as a means of immune evasion, a tactic shared by other viruses such as SARS-CoV-2, Lassa virus (98), and Ebola virus (99). Further, within the list of IGHV4–34 antibody structures compiled in this review (**Supplementary Table S1**), all these viruses are represented as targets (multiple for HIV and SARS-CoV-2), many with an expanded HCDR3 explained as a requisite for glycan shield penetration. The hydrophobic patch, in effect, may represent an entry point (a foot in the door, so to speak) towards a humoral response for glycan-heavy targets, explaining the prevalence of this germline class in both HIV and SARS-CoV-2 infection, and justifying maintenance of the IGHV4–34 germline in spite of the inherent disadvantage of self-reactivity noted in pathologies such as CAD and CAS.
