# Supplementary material for: Framework-mediated binding of foreign and self-glycans by IGHV4–34 antibodies
Source: Front Immunol. 2026 Feb 18;17:1767837. doi: 10.3389/fimmu.2026.1767837 (PMC12956792; doi:10.3389/fimmu.2026.1767837)
Supplement: Supplementary file 1 [file DataSheet1.docx]

**Framework-mediated binding of foreign and self-glycans by IGHV4-34 antibodies**

**Langley et al., (2026) Frontiers in Immunology**

**Supplementary Information**


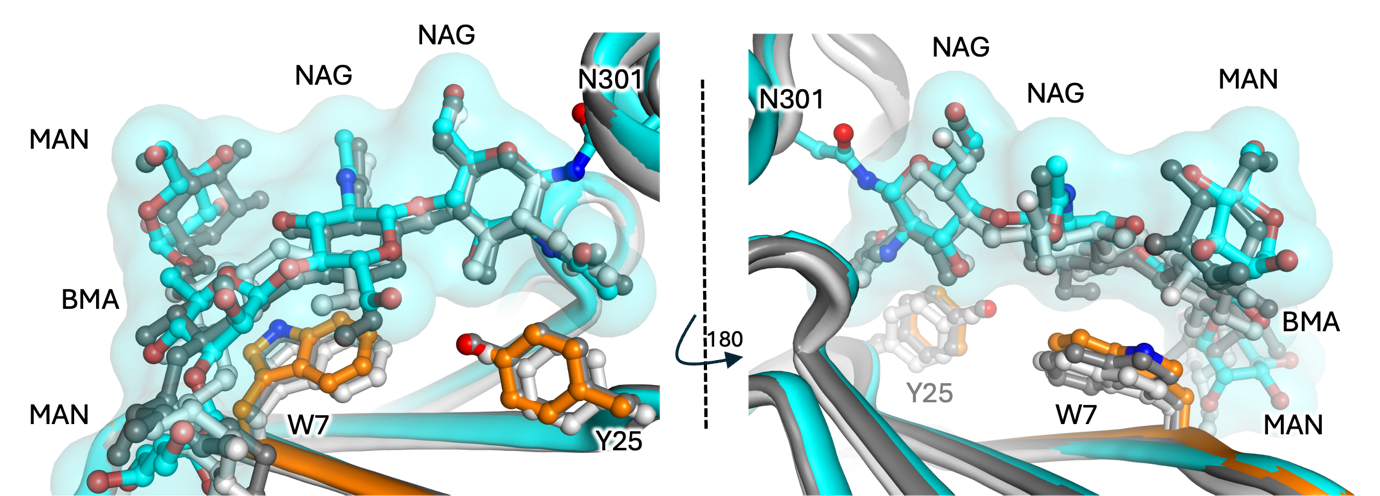


**Figure S1**. Superposition of the three ACS114 interfaces within the GP120 complex. Superposition was about the GP120 component. The complex presented in the main text is colored cyan (GP120, with surface about the N301 carbohydrate) and orange (ACS114 antibody heavy chain). The other superposed complexes are colored light and dark grey, respectively.
