# Supplementary material for: Framework-mediated binding of foreign and self-glycans by IGHV4–34 antibodies
Source: Front Immunol. 2026 Feb 18;17:1767837. doi: 10.3389/fimmu.2026.1767837 (PMC12956792; doi:10.3389/fimmu.2026.1767837)
Supplement: Supplementary file 2 [file Table1.docx]

**Framework-mediated binding of foreign and self-glycans by IGHV4-34 antibodies**

**Langley et al., (2026) Frontiers in Immunology**

**Supplementary Information**

**Table S1**. PDB depositions containing IGHV4-34 antibodies. PDB entries containing IGHV4-34 antibodies are listed chronologically and include method, resolution, scientific context, ligand (if present), status of residues W7 and Y25, VL pairing, HCDR3 length, general comment regarding interface, and reference. NL indicates no ligand, Std indicates either standard poise of W7 and Y25 (side chains projected towards each other) or a conventional antibody-ligand interface. HCDR3 length (#H3) is based on the International Immunogenetics (IMGT) delineation as determined using the online tool AbRSA (AbRSA: A robust tool for antibody numbering (https://doi.org/10.1002/pro.3633) 2019, Protein Science). Lengths ≥ 20 are emboldened and noted as “long H3” in the comment. Entries highlighted yellow contain interfaces between the FR1 hydrophobic patch and ligand-projected glycans. Entries highlighted grey contain FR1 mutations at positions 7 or 25. Entries highlighted mauve contain Rhesus Macaque antibodies of germline IGHV4-73.

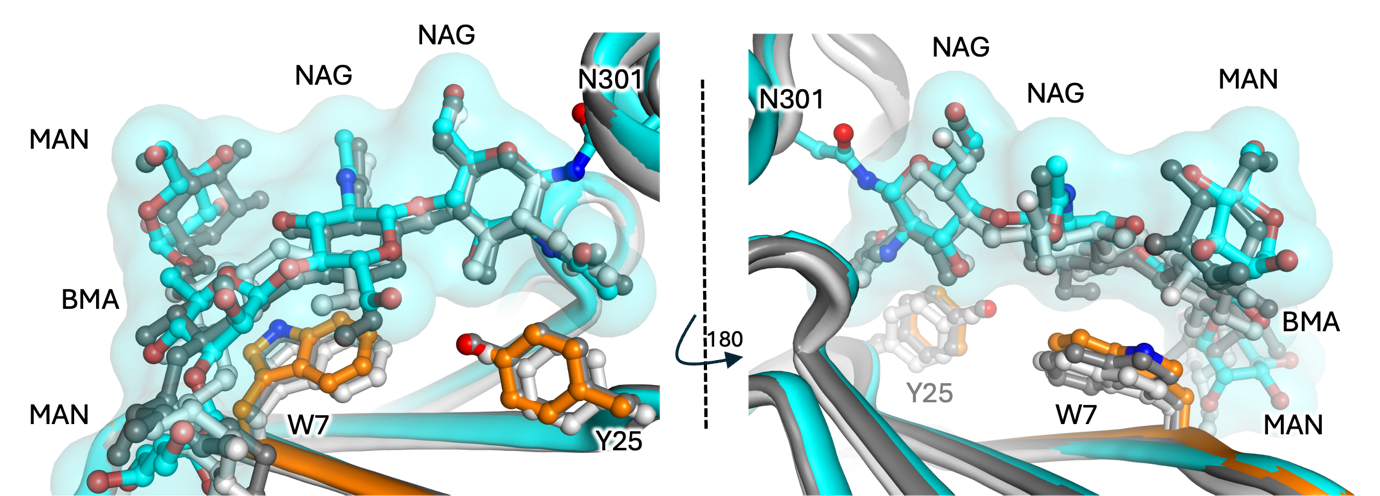


**Figure S1**. Superposition of the three ACS114 interfaces within the GP120 complex. Superposition was about the GP120 component. The complex presented in the main text is colored cyan (GP120, with surface about the N301 carbohydrate) and orange (ACS114 antibody heavy chain). The other superposed complexes are colored light and dark grey, respectively.
